# AZI2 mediates TBK1 activation at unresolved selective autophagy cargo receptor complexes with implications for CD8 T-cell infiltration in breast cancer

**DOI:** 10.1080/15548627.2023.2259775

**Published:** 2023-09-21

**Authors:** Syn Kok Yeo, Michael Haas, Kanakaraju Manupati, Mingang Hao, Fuchun Yang, Song Chen, Jun-Lin Guan

**Affiliations:** a Department of Cancer Biology, University of Cincinnati College of Medicine, Cincinnati, OH, USA; b Translational Research Institute, Henan Provincial People’s Hospital, Academy of Medical Science, Zhengzhou University, Zhengzhou, Henan Province, China

**Keywords:** AZI2, breast cancer, RB1CC1, immune checkpoint inhibitor, TBK1

## Abstract

Most breast cancers do not respond to immune checkpoint inhibitors and there is an urgent need to identify novel sensitization strategies. Herein, we uncovered that activation of the TBK-IFN pathway that is mediated by the TBK1 adapter protein AZI2 is a potent strategy for this purpose. Our initial observations showed that RB1CC1 depletion leads to accumulation of AZI2, in puncta along with selective macroautophagy/autophagy cargo receptors, which are both required for TBK1 activation. Specifically, disrupting the selective autophagy function of RB1CC1 was sufficient to sustain AZI2 puncta accumulation and TBK1 activation. AZI2 then mediates downstream activation of DDX3X, increasing its interaction with IRF3 for transcription of pro-inflammatory chemokines. Consequently, we performed a screen to identify inhibitors that can induce the AZI2-TBK1 pathway, and this revealed Lys05 as a pharmacological agent that induced pro-inflammatory chemokine expression and CD8^+^ T cell infiltration into tumors. Overall, we have identified a distinct AZI2-TBK1-IFN signaling pathway that is responsive to selective autophagy blockade and can be activated to make breast cancers more immunogenic.

**Abbreviations:** AZI2/NAP1: 5-azacytidine induced 2; CALCOCO2: calcium binding and coiled-coil domain 2; DDX3X: DEAD-box helicase 3 X-linked; FCCP: carbonyl cyanide p-triflouromethoxyphenylhydrazone; a protonophore that depolarizes the mitochondrial inner membrane; ICI: immune checkpoint inhibitor; IFN: interferon; NBR1: NBR1 autophagy cargo receptor; OPTN: optineurin; RB1CC1/FIP200: RB1 inducible coiled-coil 1; SQSTM1/p62: sequestosome 1; TAX1BP1: Tax1 binding protein 1; TBK1: TANK binding kinase 1

## Introduction

ICIs (immune checkpoint inhibitors) can induce durable therapeutic responses in cancer patients [1,2]. However, the applicability of ICIs in breast cancer has been limited to triple negative breast cancer (TNBC) and ERBB2/HER2^+^ breast cancer subtypes [3–6]. As such, it remains a formidable challenge to expand the prominent benefits of this treatment modality to most breast cancer patients with tumors that are not immunogenic (i.e., luminal subtype breast cancers). To overcome this problem, there is a need to identify strategies that can stimulate immunologically quiescent or “cold” tumors into immunologically “hot” tumors, with increased tumor infiltrating lymphocytes (TILs) and improved responses to ICIs [5,7].

One approach to make “cold” tumors “hot” is through activating TBK1, a key signaling node in the IFN (interferon) pathway that is normally triggered by the presence of pathogens [8–12]. Current efforts to activate TBK1 in tumors are based on viral mimicry strategies with agonists that can activate typical TBK1 adaptor proteins such as STING1 and MAVS [10,13–16]. These adapters normally function as sensors for cytoplasmic DNA and viral RNA respectively, but in the presence of therapeutic agonists, they can also promote TBK1 auto-phosphorylation by inducing higher order TBK1 oligomerization [8,17,18]. Subsequently, TBK1 activation leads to phosphorylation of downstream transcription factors of the interferon response such as IRF3 and IRF7, along with expression of pro-inflammatory chemokines [19,20]. Hence, the use of STING1 and MAVS agonists represent a general viral mimicry strategy that can be potentially exploited to activate the TBK1-IFN pathway for improved ICI responses [10,14–16]. However, tumors can develop resistance mechanisms by silencing these adapter proteins [21] and it would be beneficial to uncover alternative strategies for this purpose.

Macroautophagy/autophagy is a lysosomal degradation pathway utilized by cells to degrade cargo that are enclosed within double membrane structures termed autophagosomes, for cytoplasmic quality control or recycling of metabolites [22,23]. More specifically, autophagy can be further classified into a more generic process termed “bulk autophagy” that degrades cytoplasmic material upon nutrient deprivation and “selective autophagy” that involves degradation of specific cargo such as viruses, bacteria, or damaged organelles [24–29]. In both processes, RB1CC1/FIP200 plays a key scaffolding role to initiate autophagosome formation [30]. The N-terminal portion of RB1CC1 interacts with ATG13 and together with ULK1 and ULK2, this complex mediates nutrient sensing signals from AMPK and MTOR to regulate bulk autophagy [31–34]. Alternatively, the C-terminal domain of RB1CC1 that shares homology with yeast Atg11, plays a role in selective autophagy and can interact with selective autophagy cargo receptors such as SQSTM1/p62, CALCOCO2, TAX1BP1, CCPG1, NBR1 and OPTN [26,28,35–38]. It is also worth noting that this region of RB1CC1 interacts with the TBK1 adapter proteins AZI2/NAP1 and TBKBP1/SINTBAD [38,39]. Moreover, TBK1 has been shown to be important in selective degradation of mitochondria (mitophagy), by phosphorylating the cargo receptors SQSTM1, OPTN and CALCOCO2 [28,40–42], highlighting the role of this kinase in the regulation of selective autophagy. Recent observations also suggest that selective autophagy can proceed in an LC3-lipidation independent manner, albeit less efficiently [35,43,44]. Thus, it is possible that RB1CC1’s C terminus can function independently from its N terminus that is crucial for instigating downstream processes involving LC3-lipidation machinery (e.g., ATG12–ATG5-ATG16L1).

More recently, it has been found that depletion of RB1CC1 can lead to TBK1 hyper-activation [45,46]. Moreover, we have found that the activation of TBK1 upon loss of RB1CC1 can promote CD8^+^ T cell infiltration into mammary tumors and sensitize them to ICIs [39]. However, the mechanisms that orchestrate TBK1 activation in the absence of RB1CC1 remains unclear and it is important to elucidate this process to uncover novel therapeutic strategies and agents that can sensitize breast cancers to ICIs. In this study, we uncovered a key role for the TBK1 adapter protein, AZI2, in mediating TBK1 activation at unresolved selective autophagy receptor complexes upon depletion of RB1CC1. Furthermore, we identified Lys05 as a therapeutic agent that can induce TBK1 activation, leading to increased pro-inflammatory cytokine expression, interferon responses and CD8^+^ T cell recruitment. These findings establish a new parallel for TBK1-IFN pathway activation that is mediated by AZI2 and can be triggered by disruption of selective autophagy.

## Results

### RB1CC1 ablation leads to the formation of AZI2 puncta that colocalize with selective autophagy cargo receptors

TBK1 adapter proteins play a key role in the auto-activation of TBK1 by promoting higher order oligomerization of this kinase [17,18]. In order to understand the underlying mechanism for TBK1 activation upon depletion of RB1CC1, we have previously identified AZI2 as the key adapter protein that is essential for TBK1 hyper-activation under this circumstance [39]. We have also excluded the requirement of other TBK1 adapter proteins, TBKBP1 and TANK, in our prior study and have now found that other interferon pathway related TBK1 adapters STING1 and MAVS were not enriched upon depletion of RB1CC1 by CRISPR in MDA-MB-231 breast cancer cells (Fig. S1A). This indicated that TBK1 activation in RB1CC1-deficient cells was not due to accumulation of STING1 or MAVS upon autophagy blockade [47,48]. To further validate this, we depleted STING1 and MAVS by siRNA in 231 cells with *RB1CC1* KO, but neither of these perturbations diminished p-TBK1 (S172) levels (Fig. S1B). Altogether, this provided justification for examining AZI2 as a key player in TBK1 activation upon depletion of RB1CC1.

**Figure 1.**
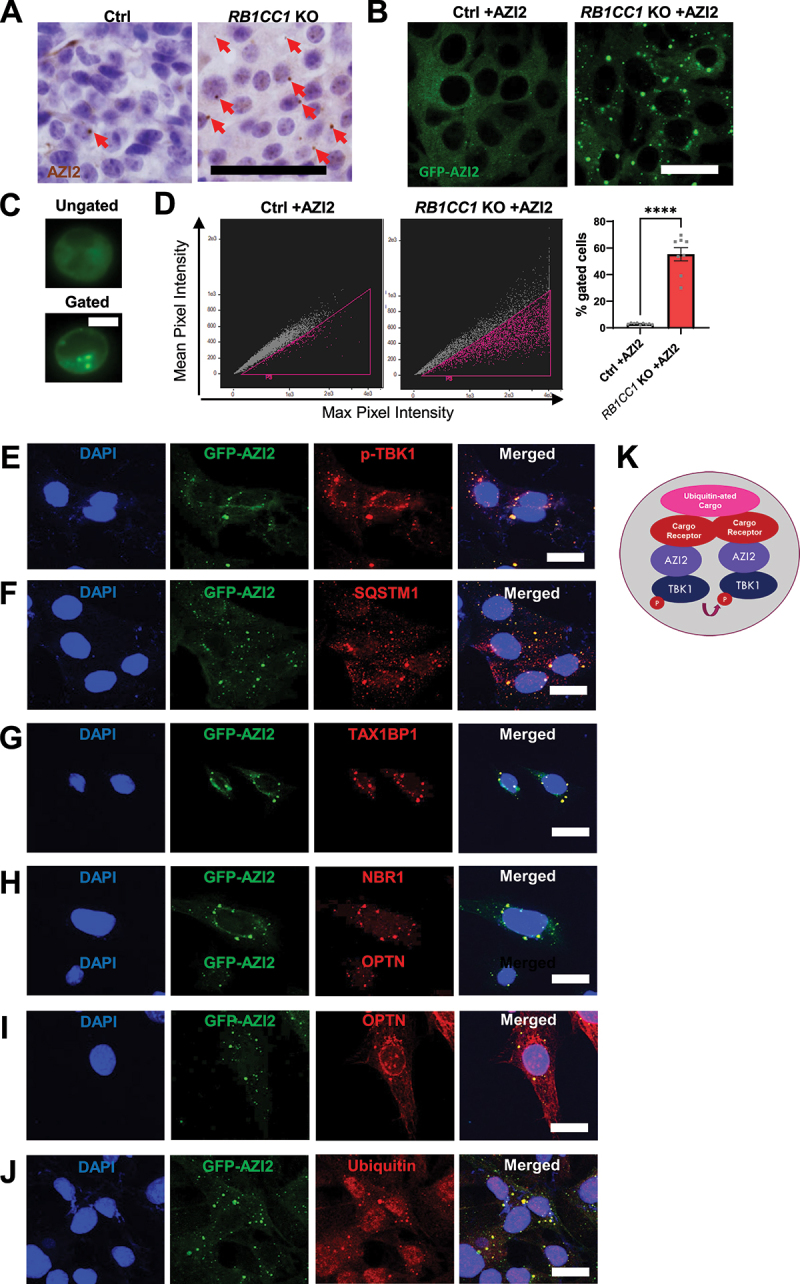
RB1CC1 ablation leads to the formation of AZI2 puncta. (A) micrographs showing immunohistochemical staining of AZI2 puncta (red arrows) in transplanted Ctrl or *RB1CC1* KO tumors. Scale bar: 100 µm. (B) confocal imaging of GFP-AZI2 in Ctrl or *RB1CC1* KO +AZI2 cells. Scale bar: 20 µm. (C) representative images for events gated from imaging cytometry analysis in D. Scale bar: 7 µm. (D) dot plots showing mean pixel intensity against max pixel intensity from imaging cytometry analysis of Ctrl or *RB1CC1* KO +AZI2 cells. Bar chart shows percentage gated cells representing cells with GFP-AZI2 puncta. ****indicates *p* < 0.0001. (E-I). Confocal imaging of *RB1CC1* KO +AZI2 cells showing colocalization of GFP-AZI2 with cells stained for (E) p-TBK1, (F) SQSTM1, (G) TAX1BP1, (H) NBR1, (I) OPTN and (J) ubiquitin. Scale bar: 20 µm. (K) model depicting the constituents colocalizing with GFP-AZI2 in puncta.

One key feature that is associated with TBK1 adapter proteins upon activation is the change in their localization or oligomerization, such as the translocation of STING1 from the ER membrane to the Golgi [17]. Interestingly, when we examined tissue sections from transplanted polyoma middle T (PyMT) driven mammary tumors (designated as Ctrl cells) that can be induced to conditionally ablate RB1CC1 upon tamoxifen administration (designated as *RB1CC1* KO cells) [39], we found that tumors formed by *RB1CC1* KO cells displayed punctate AZI2 staining, while tumors formed by Ctrl cells exhibited mostly diffuse staining of AZI2 (Figure 1A). To validate the *in vivo* observations, we took advantage of PyMT: sgAZI2 mammary tumor cells that we have generated previously [39] and re-expressed GFP-AZI2 in these cells, to allow for visualization of AZI2 localization in live cells, without the need for antibody staining (PyMT: sgAZI2: GFP-AZI2 cells, abbreviated as Ctrl +AZI2 cells and *RB1CC1* KO +AZI2, before and after hydroxy-tamoxifen induced deletion of *RB1CC1*, respectively). By comparing Ctrl +AZI2 and *RB1CC1* KO +AZI2 cells, it was evident that AZI2 formed punctate structures in the absence of RB1CC1 (Figure 1B). By establishing this *in vitro* system, we could also subject these cells expressing GFP-AZI2 to imaging flow cytometry (gating strategy depicted in Fig. S1C), to allow for high-throughput unbiased quantification of AZI2 puncta in cells. This orthogonal method also revealed a stark increase in the percentage of cells with AZI2 puncta upon depletion of RB1CC1 (Figures. 1C,D). These rigorous observations set the foundation for examining the novel phenomena of AZI2 puncta formation upon depletion of RB1CC1.

To understand these AZI2 punctate structures in more detail, we performed co-immunoprecipitation experiments of GFP-AZI2 with lysates from Ctrl +AZI2 and *RB1CC1* KO +AZI2 cells respectively. The co-immunoprecipitated proteins from each cell type was then subjected to liquid chromatography-mass spectrometry (LC-MS) (Supplementary Table 1). A network analysis of proteins enriched in *RB1CC1* KO +AZI2 cells, that have increased puncta formation, was then performed using STRING analysis [49]. Interestingly, this analysis revealed that AZI2 was interacting with selective autophagy cargo receptors such as TAX1BP1, NBR1 and SQSTM1 along with known interactors such as TBK1 (Fig. S1D). Crucially, when we performed immuno-fluorescence experiments in *RB1CC1* KO +AZI2 cells, we could observe activated p-TBK1 (S172) staining that coincided with the locations of GFP-AZI2 puncta (Figure 1E). This indicated that AZI2 puncta were indeed the sites of TBK1 activation. Notably, colocalization of GFP-AZI2 with SQSTM1, TAX1BP1, NBR1 and OPTN reaffirms the mass spectrometry results (Figures. 1F–I), whereas only partial colocalization was observed for CALCOCO2 (Fig. S1E). Both AZI2 and TBK1 have been implicated in the process of selective autophagy [26] and the presence of multiple selective autophagy cargo receptors at GFP-AZI2 puncta were in line with their cooperative functions during this process [36]. These selective autophagy receptors are usually recruited to ubiquitinated cargoes and indeed we observed colocalization of GFP-AZI2 with ubiquitin as well (Figure 1J). We also inspected colocalization with other key organelle markers such as the endoplasmic reticulum (PDIA4/ERP72), Golgi complexes (GOLGA1/Golgin97), endosomes (RAB5A, RAB7A), lysosomes (LAMP1), mitochondria (TOMM20) and autophagosomes (MAP1LC3B) but did not find substantial overlap between AZI2 and these organelle markers (Fig. S1E). Overall, we have found the formation of AZI2 punctate structures that are associated with activated TBK1, selective autophagy cargo receptors and ubiquitin upon depletion of RB1CC1 (Figure 1K). This suggested that blockade of selective autophagy could lead to accumulation of unresolved AZI2-TBK1 signaling complexes.

### AZI2 puncta formation is required for TBK1 activation and is downstream of cargo receptor aggregation

**Figure 2.**
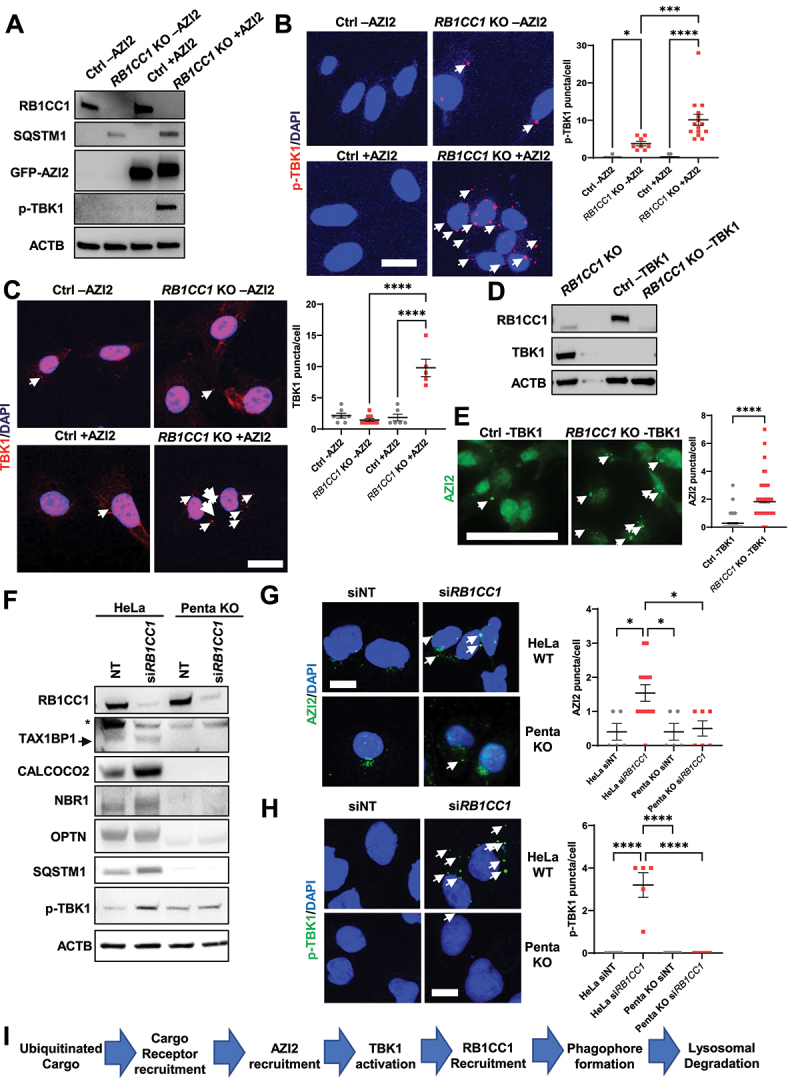
AZI2 puncta formation is required for TBK1 activation and is downstream of cargo receptor aggregation. (A) immunoblots showing levels of RB1CC1, SQSTM1, GFP-AZI2, p-TBK1 and ACTB in Ctrl or *RB1CC1* KO cells ± AZI2 expression. (B) confocal imaging showing formation of p-TBK1 puncta (white arrows) in Ctrl or *RB1CC1* KO cells ± AZI2 expression. Scale bar: 10 µm. Bar chart shows quantification of p-TBK1 puncta per cell for each condition. (C) confocal imaging showing formation of TBK1 puncta (white arrows) in Ctrl or *RB1CC1* KO cells ± AZI2 expression. Scale bar: 10 µm. Bar chart shows quantification of TBK1 puncta per cell for each condition. (D) immunoblots showing levels of RB1CC1, TBK1 and ACTB in Ctrl or *RB1CC1* KO ± TBK1 expression. (E) confocal imaging showing formation of AZI2 puncta (white arrows) in Ctrl or *RB1CC1* KO cells ± TBK1 expression. Scale bar: 50 µm. (F) immunoblots showing levels of RB1CC1, TAX1BP1, CALCOCO2, NBR1, OPTN, SQSTM1, p-TBK1 and ACTB in HeLa or Penta KO cells treated with non-targeting or siRNA against *RB1CC1*. (G-H) confocal imaging showing formation of (G) AZI2 or (H) p-TBK1 puncta (white arrows) in HeLa or Penta KO cells treated with non-targeting or siRNA against *RB1CC1*. Scale bar: 10 µm. Bar charts showing quantification of puncta/cell. * indicates *p* < 0.05, ****indicates *p* < 0.0001. (I) flow chart showing sequence of key events related to TBK1 activation in selective autophagy.

Upon identification of some of the molecular constituents within AZI2 puncta, we were then interested in dissecting the sequence by which these components were recruited to these protein complexes. Using PyMT: sgAZI2 mammary tumor cells [39] with re-expression of GFP (designated as Ctrl -AZI2 and *RB1CC1* KO -AZI2 cells, before and after hydroxy-tamoxifen induced deletion of *RB1CC1*, respectively), we found that ablation of RB1CC1 did not lead to TBK1 activation in *RB1CC1* KO -AZI2 (Figure 2A). Contrastingly, in *RB1CC1* KO +AZI2 cells, there was induction of TBK1 activation upon RB1CC1 depletion (Figures. 2A,B), reiterating a key role for AZI2 in this mode of TBK1 activation. Apart from its activation (p-TBK1 levels), in contrast to *RB1CC1* KO +AZI2 cells, the recruitment of TBK1 into punctate structures was also abolished in *RB1CC1* KO -AZI2 cells (Figure 2C). In the case of cargo receptor puncta formation, *RB1CC1* KO -AZI2 cells maintained the formation of SQSTM1, TAX1BP1, NBR1 and OPTN puncta (Fig. S2A), suggesting that AZI2 does not function upstream of cargo receptor accumulation. Additionally, we found that cells depleted of TBK1 (−TBK1) still exhibited increased AZI2 puncta formation upon loss of RB1CC1 (Figures. 2D,E). Altogether, this indicated that AZI2 recruitment into punctate structures is a key event that occurs upstream of TBK1 activation, but likely downstream of selective autophagy cargo receptor complex formation.

To determine whether selective autophagy cargo receptor accumulation were indeed upstream events relative to AZI2 recruitment and TBK1 activation, we utilized HeLa cells depleted of *TAX1BP1*, *CALCOCO2*, *NBR1*, *OPTN* and *SQSTM1* (Penta KO cells) that were generated by Lazarou et el [42]. In HeLa cells, silencing of RB1CC1 led to increased p-TBK1 levels (Figure 2F, first 2 lanes), whereas this response was attenuated in the Penta KO cells (Figure 2F, Lanes 3–4). Similarly, we also found by immuno-fluorescence analyses that the AZI2 and p-TBK1 puncta formation that occurs upon silencing of *RB1CC1* in HeLa cells, was abolished in Penta KO cells (Figures. 2G,H). In RB1CC1^F/F^: SQSTM1^−/−^ cells [50], when we depleted RB1CC1 by adenovirus mediated Cre delivery (Ad-Cre), p-TBK1 and AZI2 puncta formation could still proceed (Figs. S2B-C). It is likely that the other cargo receptors could function redundantly in the absence of SQSTM1 alone since we do observe formation of TAX1BP1 puncta in these SQSTM1 depleted cells (Fig. S2C). Altogether, our genetic analyses of the components within AZI2 puncta indicate that aggregation of selective autophagy cargo receptors into puncta precedes AZI2 recruitment, which then leads to TBK1 activation (Figure 2I). Given the known functions of RB1CC1 and these other constituents in the process of selective autophagy [24], it is possible that the posited sequence of events occur prior to RB1CC1 recruitment and phagophore formation. Thus, when RB1CC1 is genetically depleted, selective autophagy is stalled, leading to the accumulation of these upstream components that are normally recruited prior to the formation of the phagophore (Figure 2I).

### AZI2 puncta formation and TBK1 activation are markedly induced upon inhibition of selective autophagy

**Figure 3.**
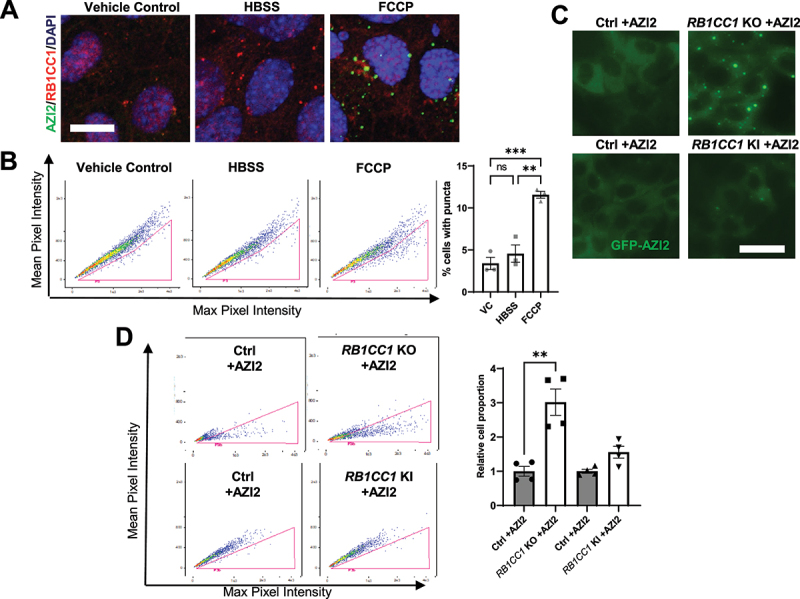
AZI2 puncta formation and TBK1 activation are markedly induced upon inhibition of selective autophagy. (A) confocal imaging showing AZI2 (green) and RB1CC1 (red) puncta formation in Ctrl +AZI2 cells cultured under control, HBSS conditions or 20 µM FCCP treatment for 3 h. Scale bar: 10 µm. (B) dot plots from imaging cytometry analysis of Ctrl +AZI2 cells cultured under control, HBSS conditions or 20 µM FCCP treatment for 3 h. Bar charts showing quantification of percentage cells with GFP-AZI2 puncta. ***indicates *p* < 0.001, **indicates *p* < 0.01, ns indicates not significant. (C) fluorescence microscopy imaging of GFP-AZI2 puncta in Ctrl or *RB1CC1* KO or *RB1CC1* KI cells +AZI2. Scale bar: 25 µm. (D) dot plots from imaging cytometry analysis of cells in C. Bar charts showing quantification for relative percentage of cells with GFP-AZI2 puncta. **indicates *p* < 0.01.

If indeed AZI2 were recruited to puncta containing cargo receptors at the initial steps of selective autophagy, we should also be able to observe some level of AZI2 puncta formation upon induction of selective autophagy, even without deletion of RB1CC1 leading to further accumulation of AZI2 puncta. To investigate this, Ctrl +AZI2 cells were treated with HBSS (Hank’s buffered salt solution) or 20 µM FCCP (trifluoromethoxy-carbonylcyanide-phenylhydrazone) for 3 h to induce bulk or selective autophagy of mitochondria (mitophagy) respectively (Figure 3A). Interestingly, the formation of GFP-AZI2 puncta was prominent in FCCP treated cells but not HBSS treated cells, whereas RB1CC1 puncta formation could be observed under both conditions (Figure 3A). This observation was recapitulated using imaging cytometry assays, with a higher percentage of cells containing GFP-AZI2 puncta in FCCP-treated cells relative to control but not when bulk autophagy was induced by HBSS (Figure 3B). In contrast, by comparing Ctrl -AZI2 and *RB1CC1* KO -AZI2 cells which express GFP only, no increase in puncta formation were observed by microscopy nor imaging flow cytometry (Figs. S2D-E). Moreover, neither HBSS treatment nor FCCP treatment induced puncta formation in Ctrl -AZI2 cells, indicating that the puncta forming properties observed in Ctrl or *RB1CC1* KO +AZI2 cells were due to AZI2 and not GFP (Fig. S2F). In addition to FCCP treatment to induce mitophagy, we could also observe GFP-AZI2 puncta formation upon treatment with other agents (i.e., MG132 and H_2_O_2_) that are known to induce other forms of selective autophagy (Fig. S3A). With FCCP treated Ctrl +AZI2 cells, we were also able to observe the kinetics of AZI2 puncta formation and TAX1BP1 puncta formation in more detail (Fig. S3B). Interestingly, we could observe increased TAXBP1 puncta per cell at 60 minutes post-FCCP treatment timepoints but increased GFP-AZI2 puncta were only observed at 120 minutes post-FCCP treatment (Fig. S3B). This observation supports the model of selective autophagy cargo receptor aggregation preceding AZI2 recruitment (Figure 2I). In the case of FCCP induced AZI2 puncta formation, it is also interesting to note that this stimulus for mitophagy induced AZI2 puncta that coincided with TAX1BP1 and OPTN, but not SQSTM1 (Fig. S3C). This suggests that there could still be specificity in the involvement of cargo receptors at the early stages of selective autophagy, with respect to different cargoes or stimuli. Altogether, this suggested that AZI2 puncta formation is generally induced in various forms of selective autophagy but not starvation induced bulk autophagy.

**Figure 4.**
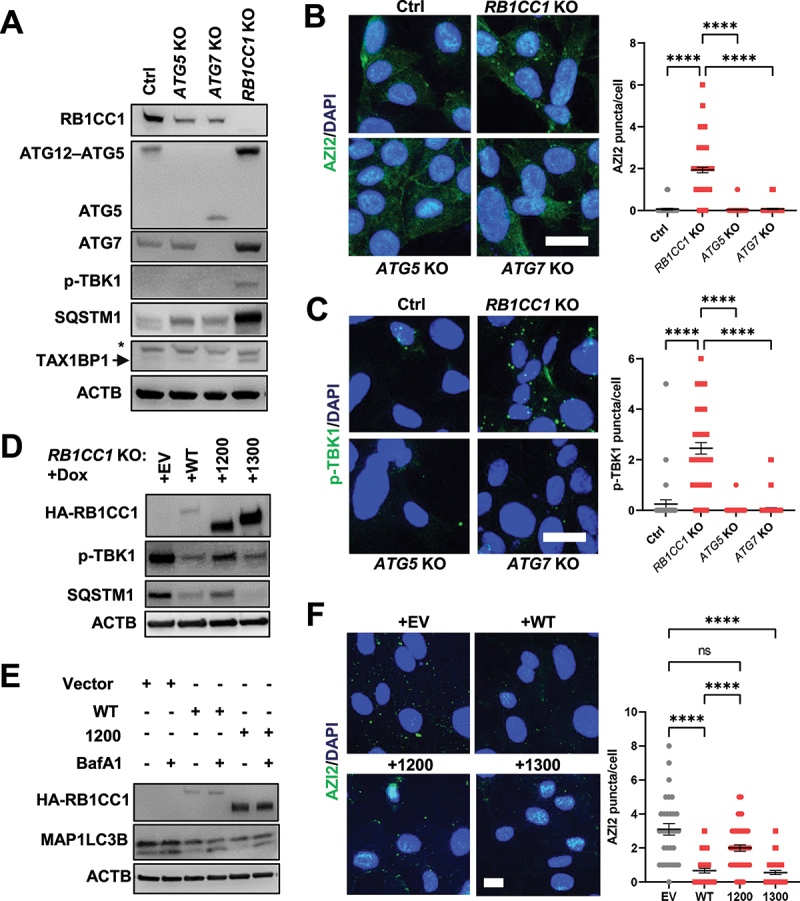
Depletion of genes required for selective autophagy induces AZI2 puncta and TBK1 activation. (A) immunoblots showing levels of RB1CC1, ATG5, ATG7, p-TBK1, SQSTM1, TAX1BP1 and ACTB in Ctrl, *RB1CC1* KO, *ATG5* KO and *ATG7* KO cells. (B-C) confocal imaging of (B) GFP-AZI2 or (C) p-TBK1 puncta in Ctrl, *RB1CC1* KO, *ATG5* KO and *ATG7* KO cells. Bar charts showing quantification for number of puncta per cell. ****indicates *p* < 0.0001. Scale bar: 20 µm. (D) immunoblots showing levels of HA-RB1CC1, p-TBK1, SQSTM1 and ACTB in *RB1CC1* KO cells transduced with doxycycline inducible empty vector (+EV), RB1CC1 WT (+WT), RB1CC1 residues 1–1200 (+1200) or 1–1300 (+1300). (E) immunoblots showing levels of HA-RB1CC1, MAP1LC3B or ACTB in *RB1CC1* KO cells transduced with doxycycline inducible empty vector (+EV), RB1CC1 WT (+WT) or RB1CC1 residues 1–1200 (+1200) ± 200 nM BafA1 treatment. (F) confocal imaging of GFP-AZI2 puncta in *RB1CC1* KO cells transduced with doxycycline inducible empty vector, RB1CC1 WT, RB1CC1 residues 1–1200 or RB1CC1 residues 1–1300. Scale bar: 10 µm. Bar charts showing quantification for number of puncta per cell. ****indicates *p* < 0.0001, ns indicates not significant.

RB1CC1’s N-terminus has been found to be important for LC3-lipidation and bulk autophagy in our recent study [31], while its C-terminus ATG11 homology domain has been reported to play a role in selective autophagy [26–28]. We have previously generated mice with a *RB1CC1* KI allele (mutation in residues 582–585 within RB1CC1’s N-terminus) that effectively inhibits LC3-lipidation to the extent of that observed in *RB1CC1* KO cells [31,39] but we have found that the accumulation of the selective autophagy cargo receptor, SQSTM1, is not as marked in *RB1CC1* KI cells relative to *RB1CC1* KO cells (Fig. S4A). Although, there was less SQSTM1 accumulation in *RB1CC1* KI cells, it was equivalent to that observed in ATG16L depleted cells (Fig. S4A). Since LC3-lipidation is not an absolute requirement for selective autophagy [35], the difference in SQSTM1 accumulation in *RB1CC1* KI and *RB1CC1* KO cells indicate that *RB1CC1* KI cells still harbor residual selective autophagy activity through RB1CC1’s C-terminus. Accordingly, we were interested in dissecting the contributions of these specific domains of RB1CC1 toward the regulation of AZI2 puncta formation. To this end, we utilized *RB1CC1* KI: sgAZI2: GFP-AZI2 cells that can be induced by hydroxy-tamoxifen to delete the floxed *RB1CC1* allele, leaving only the bulk-autophagy-defective *RB1CC1* KI allele [31] (abbreviated as *RB1CC1* KI +AZI2 cells; these cells before induced deletion contain one functional *RB1CC1* allele and are designated Ctrl +AZI2 cells). Interestingly, *RB1CC1* KI +AZI2 cells did not exhibit any marked increase in GFP-AZI2 puncta formation when observed by microscopy nor imaging cytometry (Figures. 3C,D) despite inhibition of LC3-lipidation in these cells [39]. Concomitantly, depletion of autophagy proteins involved in the LC3-lipidation machinery (e.g., ATG5 and ATG7) did not lead to TBK1 activation nor AZI2 puncta formation in Ctrl cells (Figure 4A–C) Recent reports have indicated that selective autophagy can occur without the LC3-lipidation machinery, albeit less efficiently [35,43,44]. This is further supported by our observation that depletion of ATG5 or ATG7 leads to less accumulation of SQSTM1 and TAX1BP1 relative to cells lacking RB1CC1 (Figure 4A), suggesting selective autophagy flux was not completely blocked upon disrupting LC3-lipidation. Thus, our results indicate that disrupting LC3-lipidation (i.e., through expression of RB1CC1 KI or knockout of ATG5, ATG7) was not sufficient to induce AZI2 puncta and TBK1 activation (Figures 3C,D and 4A–C) because selective autophagy was not sufficiently perturbed.

Conversely, depletion of ATG13, that is required for both bulk and selective autophagy [24,32,33], could induce TBK1 activation and AZI2 puncta formation to the same degree as RB1CC1-depleted cells (Figs. S4B-D). To further ascertain that blocking selective autophagy is sufficient for AZI2 puncta formation and TBK1 activation, we generated RB1CC1 C-terminal truncation mutants to specifically disrupt its selective autophagy functions (Figure 4D). *RB1CC1* KO cells were reconstituted with doxycycline-inducible empty vector (+EV), wildtype RB1CC1 (+WT), RB1CC1 residues 1–1200 (+1200), or residues 1–1300 (+1300). As expected, re-expression of WT RB1CC1 could rescue the increased p-TBK1 and SQSTM1 levels and the same can be observed for re-expression of RB1CC1 residues 1–1300 (Figure 4D, Fig. S4E). However, re-expression of RB1CC1 residues 1–1200 which lack both the coiled-coil regions that bind to some selective autophagy receptors such as TAX1BP1, and the claw domain of RB1CC1 (Fig. S4F-G), was deficient in rescuing elevated p-TBK1 and SQSTM1 levels. Notably, the MAP1LC3B flux was still similar for cells with re-expression of WT and RB1CC1 residues 1–1200 (Figure 4E), indicating that residues 1–1200 of RB1CC1 maintains bulk autophagy functions in the absence of its selective autophagy function. Consistent with the effects on p-TBK1 levels (Figure 4D, Fig. S4E), we found that re-expression of WT and RB1CC1 residues 1–1300, but not RB1CC1 residues 1–1200, could reduce AZI2 puncta formation in *RB1CC1* KO cells (Figure 4F). Taken together, these results indicate that inhibition of selective autophagy can contribute to the formation of AZI2 puncta and TBK1 activation, while residues 1200–1300 of RB1CC1 appear to be critical for mediating this function.

### DDX3X interacts with AZI2 and contributes to the expression of pro-inflammatory genes upon TBK1 activation in RB1CC1 depleted cells

**Figure 5.**
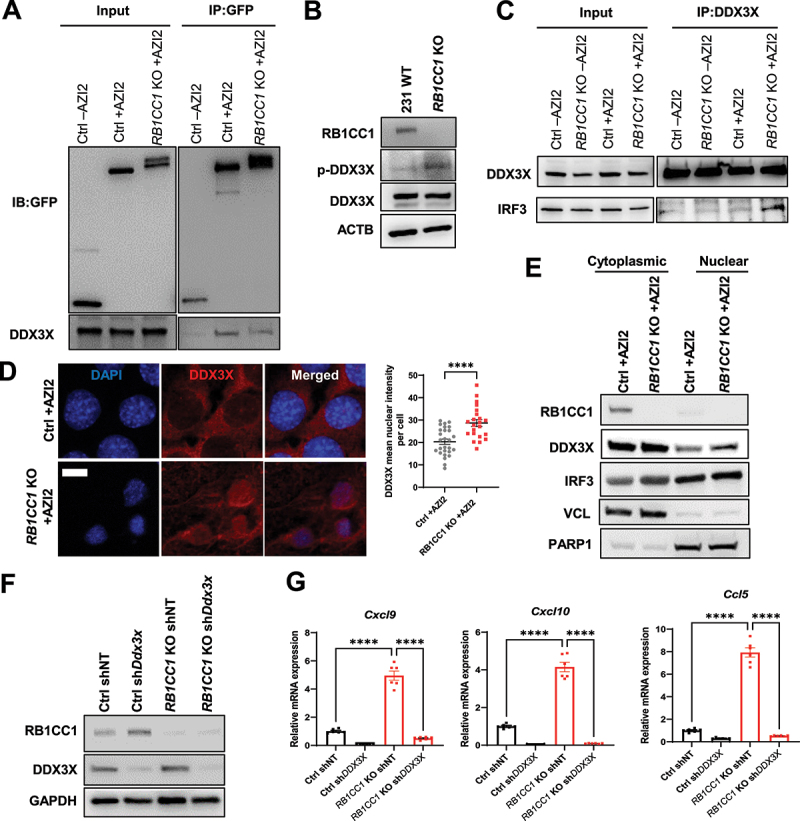
DDX3X interacts with AZI2 and contributes to the expression of pro-inflammatory genes upon TBK1 activation in RB1CC1 depleted cells. (A) immunoblots of GFP and DDX3X showing levels of these proteins in inputs and IP:GFP fractions from Ctrl -AZI2, Ctrl +AZI2 and *RB1CC1* KO +AZI2 cells. (B) immunoblots showing levels of RB1CC1, p-DDX3X (S102), DDX3X and ACTB in MDA-MB-231 WT and *RB1CC1* KO cells. (C) immunoblots of DDX3X and IRF3 showing levels of these proteins in inputs and IP:DDX3X fractions from Ctrl -AZI2, *RB1CC1* KO -AZI2, Ctrl +AZI2 and *RB1CC1* KO +AZI2 cells. (D) confocal imaging of GFP-AZI2 puncta and DDX3X localization in Ctrl or *RB1CC1* KO +AZI2 cells. Scale bar: 10 µm. (E) immunoblots showing levels of RB1CC1, DDX3X, IRF3, VCL and PARP1 in cytoplasmic and nuclear extracts from Ctrl or *RB1CC1* KO +AZI2 cells. (F) immunoblots showing levels of RB1CC1, DDX3X and GAPDH in Ctrl or *RB1CC1* KO +AZI2 cells. (G) bar charts showing relative mRNA levels of *Cxcl9, Cxcl10* and *Ccl5* normalized to *Actb* expression in Ctrl or *RB1CC1* KO +AZI2 cells and respective shRnas.

Upon TBK1 activation, adapter proteins such as AZI2 also play key roles in bridging downstream substrates with TBK1 to induce their phosphorylation [51]. In the TBK1-IFN pathway, typical substrates include the transcription factors IRF3 or IRF7 [19,52]. Accordingly, we then investigated the levels of IRF3 and IRF7 in nuclear fractions of *RB1CC1* KO cells. Although basal levels of nuclear IRF3 and IRF7 were observed in Ctrl cells, we did not observe any increased nuclear accumulation of either of these transcription factors upon RB1CC1 depletion (Fig. S5A). To identify potential substrates of TBK1 that could impinge on the IFN pathway, we revisited the mass spectrometry data of AZI2 interacting proteins that play a role in innate immunity (Proteins highlighted in blue, Fig. S1D). Interestingly, we identified DDX3X as an AZI2 interacting protein, that has been reported to be phosphorylated by TBK1, leading to its increased association with IRF3 to promote IFN-related gene expression [53–55]. We validated the association between AZI2 and DDX3X by co-immunoprecipitation, but the interaction between these two proteins (Figure 5A) and total levels of DDX3X (Fig. S5B) were not increased upon RB1CC1 ablation. Nonetheless, it is possible that the activation of TBK1 specifically in RB1CC1-deficient cells could lead to increased phosphorylation of DDX3X by TBK1. Indeed, we observed higher levels of p-DDX3X (S102) [56] in 231 *RB1CC1* KO cells relative to wildtype MDA-MB-231 cells (Figure 5B). We also observed an increased interaction between DDX3X and IRF3 in *RB1CC1* KO +AZI2 cells relative to Ctrl +AZI2 cells and this effect was absent in *RB1CC1* KO -AZI2 cells (Figure 5C). Moreover, increased nuclear DDX3X levels were found in *RB1CC1* KO +AZI2 cells compared to Ctrl +AZI2 cells (Figures. 5D,E) and *RB1CC1* KO MDA-MB-231 cells relative to wildtype cells (Fig. S5C). These data suggest that AZI2 constitutively interacts with DDX3X but upon TBK1 activation, AZI2 promotes DDX3X phosphorylation. To validate the involvement of DDX3X in the regulation of pro-inflammatory chemokine and IFN gene expression upon loss of RB1CC1, we then generated Ctrl sh*Ddx3x* cells, which were depleted of DDX3X (Figure 5F). Silencing of *Ddx3x* could abolish the increased expression of pro-inflammatory chemokines such as *Cxcl9*, *Cxcl10* and *Ccl5* in *RB1CC1* KO cells (Figure 5G). Similarly, *DDX3X* knockdown by siRNA could reverse the increased expression of the chemokine *CCL5* upon RB1CC1 depletion in MDA-MB-231 cells (Figs. S5D-E). Collectively, these results indicate that in addition to the activation of TBK1, AZI2 also plays an important role in the propagation of downstream pro-inflammatory signaling, at least in part through DDX3X.

### Inhibitor screen identifies Lys05 as an inducer of AZI2 puncta formation and corresponding TBK1 activation with increased pro-inflammatory cytokine expression

**Figure 6.**
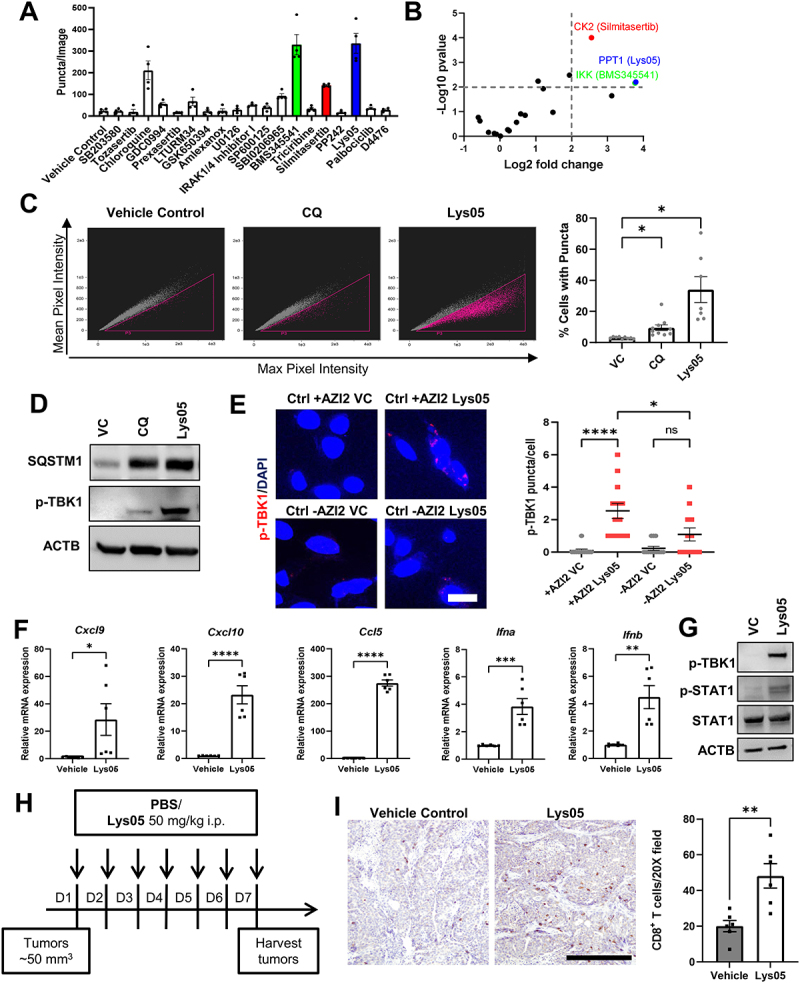
Inhibitor screen identifies Lys05 as an inducer of AZI2 puncta formation, TBK1 activation and increased CD8^+^ T-cell infiltration. (A) bar charts showing number of AZI2 puncta per image for respective inhibitors after 24-h treatment. (B) volcano plot with -log_10_ p-values and log_2_ fold change for AZI2 puncta per image for respective inhibitors relative to vehicle control treated cells. (C) dot plots from imaging cytometry analysis of Ctrl +AZI2 cells treated with vehicle control, 100 µM chloroquine (CQ) or 20 µM Lys05 for 24 h. Bar charts showing quantification of percentage cells with GFP-AZI2 puncta. *indicates *p* < 0.05. (D) immunoblots showing levels of SQSTM1, p-TBK1 and ACTB in Ctrl +AZI2 cells treated with vehicle control, 100 µM chloroquine (CQ) or 20 µM Lys05 for 24 h. (E) confocal imaging showing formation of p-TBK1 puncta in Ctrl ± AZI2 cells treated with vehicle or 20 µM Lys05. Scale bar: 10 µm. Bar chart shows quantification of p-TBK1 puncta per cell for each condition. (F) bar charts showing transcript levels of respective genes in Ctrl +AZI2 cells treated with vehicle or 20 µM Lys05. (G) immunoblots showing levels of p-TBK1, p-STAT1, STAT1 and ACTB in Ctrl +AZI2 cells treated with vehicle or 20 µM Lys05. (H) schema showing experimental design for mice transplanted with iKO cells and treated with either PBS or Lys05 at 50 mg/kg i.P. daily. (I) micrographs showing levels of CD8^+^ T cell infiltration for PBS or Lys05 treated mice. Scale bar: 200 µm. Bar chart shows quantification of CD8^+^ T cells per 20× field of view (*n* = 6 per group), **indicates *p* < 0.01.

Our results thus far have indicated that depletion of RB1CC1 leads to stalling of the selective autophagy process, resulting in accumulation of unresolved selective autophagy cargo receptor complexes that promote AZI2 mediated TBK1 activation. From our previous studies [39], we have also found that the activation of TBK1 upon depletion of RB1CC1 is important in promoting increased expression of pro-inflammatory chemokines, CD8^+^ T-cell infiltration and improved ICI responses. Although genetic depletion of *RB1CC1* can lead to the formation of AZI2 puncta and TBK1 activation, it is crucial to identify pharmacological agents to translate this prospective strategy for combination therapy with ICIs. In line with that, we employed Ctrl +AZI2 cells in a mini-screen comprising a panel of small molecule inhibitors to identify agents that could induce AZI2 puncta formation (Figure 6A). Cells were treated for 24 h with respective inhibitors and the formation of AZI2 puncta was quantified through live cell imaging. From this experiment, we identified Lys05 as an inhibitor that induced the largest fold change (13.8-fold change, *p* = 0.0063) in AZI2 puncta formation relative to vehicle treated controls at 24 h post treatment (Figure 6B). Lys05 is a dimeric chloroquine that has been shown to be a more potent autophagy inhibitor with increased anti-tumor activity [57]. The superior efficacy of Lys05 relative to chloroquine (CQ) was also recapitulated in our inhibitor screen, with chloroquine inducing only about an eight-fold change in AZI2 puncta formation (*p* = 0.0228) relative to controls (Figure 6A). Consequently, we went on to validate the ability of both these compounds to induce AZI2 puncta in Ctrl +AZI2 cells through imaging cytometry. Lys05 induced AZI2 puncta formation significantly, while CQ only generated a modest effect (Figure 6C). Accordingly, the levels of TBK1 activation upon treatment with CQ or Lys05 respectively were in line with the potency of these compounds in inducing AZI2 puncta (Figure 6D). The levels of p-TBK1 upon treatment with Lys05 were also inspected by immuno-fluorescence and we observed increased TBK1 activation in Lys05 treated Ctrl +AZI2 cells (Figure 6E, upper panels). In contrast, Ctrl -AZI2 cells did not exhibit a significant increase in p-TBK1 puncta formation (Figure 6E, lower panels), indicating that the activation of TBK1 upon Lys05 treatment occurs largely through AZI2. A key downstream feature of TBK1 activation upon depletion of RB1CC1 is the increased production of pro-inflammatory chemokines and interferons [39,58]. To assess whether Lys05 would invoke a similar response, we evaluated the expression levels of these genes and found increased *Cxcl9, Cxcl10, Ccl5, Ifna* and *Ifnb* expression in Ctrl +AZI2 cells treated with Lys05 for 72 h (Figure 6F). Moreover, increased levels of p-STAT1 were also observed upon Lys05 treatment, indicating that the TBK1-interferon pathway was activated (Figure 6G).

Consequently, we evaluated the effects of Lys05 treatment on CD8^+^ T cell infiltration in mammary tumors *in vivo*. Ctrl cells were transplanted into syngeneic FVB mice and when tumors were ~50 mm^3^ in size, they were randomized and treated with 50 mg/kg of Lys05 or vehicle as control (Figure 6H). Mice were treated with daily intraperitoneal injections for a week and tumor tissues were then harvested for immunohistochemical analysis. We observed a marked increase in tumor infiltrating CD8^+^ T cells for Lys05-treated tumors relative to vehicle controls (Figure 6I). Additionally, we also treated 4T1 cell derived tumors utilizing the same experimental design (Figure 6H) and Lys05 treatment increased CD8^+^ T cell infiltration in this model as well (Fig. S6A). Notably, the effects of pharmacologically inhibiting autophagy through Lys05 were consistent with that observed via genetic depletion of RB1CC1 [39]. To determine the importance of AZI2 in mediating CD8^+^ T cell infiltration upon autophagy inhibition, Ctrl -AZI2 cells were transplanted into mice and RB1CC1 ablation was induced by tamoxifen treatment of mice when tumors were ~50 mm^3^ (Fig. S6B), as previously described [39]. In this AZI2-deficient setting, RB1CC1 depleted tumors exhibited a comparable level of CD8^+^ T cell infiltration as controls (Fig. S6B), unlike our previous observations where RB1CC1 depletion could markedly increase CD8^+^ T cell infiltration [39]. The increased CD8^+^ T cell infiltration that is induced by Lys05 (Figure 6I) was associated with increased expression of pro-inflammatory chemokines *Cxcl9, Cxcl10* and *Ccl5* (Figure 6F) and these chemokines have been reported to play key roles in CD8^+^ T cell recruitment [59]. To determine the contribution of increased chemokines (e.g., CXCL9, CXCL10) to CD8^+^ T cell infiltration upon Lys05 treatment, mice that were treated with Lys05 were additionally administered with anti-CXCR3 blocking antibodies. By disrupting the CXCL9-CXCR3 or CXCL10-CXCR3 signaling axis, the increase in CD8^+^ T cell infiltration that was induced by Lys05 treatment can be abolished (Fig. S6C). Moreover, we inspected the expression of T cell activation and exhaustion markers for CD8^+^ T cell populations upon Lys05 treatment. We found an increase in the percentage of CD8^+^ T cells which express the activation marker CD69 but no changes for CD44 (Fig. S6D). On the other hand, CD8^+^ T cells from both vehicle or Lys05 treated tumors expressed the exhaustion marker PDCD1/CD279/PD-1 at similar levels (Fig. S6E). Altogether, these results indicate that pharmacological inhibition of autophagy via Lys05 can induce AZI2 puncta formation, TBK1 activation, downstream pro-inflammatory interferon responses and increased CD8^+^ T cell infiltration into mammary tumors.

### Increased AZI2 expression levels are associated with increased CD8^+^ T cell infiltration and better prognosis in human breast cancer patients

**Figure 7.**
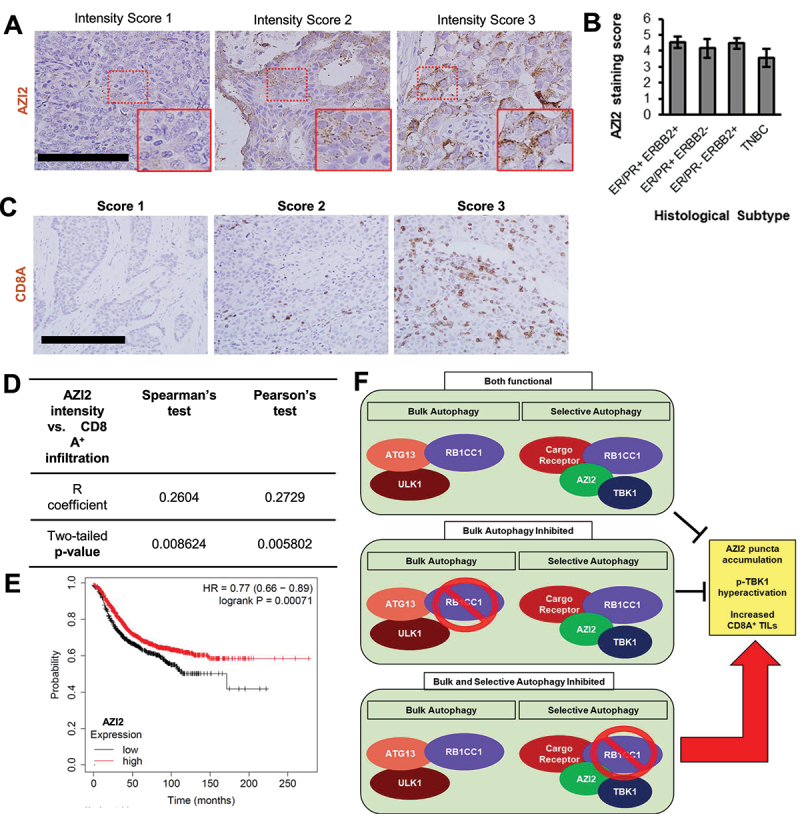
Association between AZI2 protein expression levels with CD8^+^ T cell infiltration and prognosis in human breast cancer patients. (A) micrographs showing representative images for AZI2 intensity scores by immunohistochemical analysis of a human breast cancer tissue microarray (*n* = 101). Scale bar: 100 µm. Insets show magnified areas demarcated by red boxes. (B) bar chart showing AZI2 staining scores among histological subtypes of breast cancer. No statistical significance was observed with ANOVA test (*p* = 0.48). (C) micrographs showing representative images for CD8^+^ T cell infiltration scores by immunohistochemical analysis of a human breast cancer tissue microarray (*n* = 101). Scale bar: 200 µm. (D) Supplementary table summarizing the R coefficients and p-values from Spearman’s and Pearson’s test for AZI2 intensity scores against CD8^+^ T cell infiltration scores. (E) kaplan-meier plot of relapse free survival in breast cancer patients with lowest tertile of *AZI2* gene expression (black) relative to other patients in the cohort with higher *AZI2* expression (red). Log-rank test, *p* = 0.00071. (F) graphical summary showing that inhibition of selective autophagy is necessary to elicit AZI2 puncta formation, TBK1 activation and enhanced ICI efficacy, while blocking bulk autophagy alone is not sufficient.

Based on our mechanistic studies, we have implicated AZI2 as a key adaptor protein for TBK1 activation upon inhibition of selective autophagy. We were then interested in identifying potential associations between AZI2 levels and clinical parameters in breast cancer patients. To this end, AZI2 levels were inspected in a tissue microarray of breast cancer samples (*n* = 101) by immunohistochemistry and scored in a blinded manner based on intensity and percent coverage of staining (Figure 7A). Based on this scoring, we did not find any significant changes in AZI2 scores between histological subtypes of breast cancer (Figure 7B, ANOVA test, *p* = 0.48), suggesting that AZI2 expression is not confined to particular subtypes of breast cancer. Since activation of AZI2-TBK1 signaling upon autophagy inhibition could impinge on CD8^+^ T cell infiltration (Figure 6), we then stained and scored for CD8^+^ T cell infiltration on a separate tissue microarray corresponding to the same set of samples (Figure 7C). This analysis led to an interesting positive association between AZI2 expression levels and CD8^+^ T cell infiltration in these tumors (Figure 7D), suggesting that AZI2 could play a role in human breast tumors. Furthermore, we sorted Ctrl +AZI2 cells into populations that expressed high or low GFP-AZI2 (Fig. S7A) and found that the levels of GFP-AZI2 was proportionate to the amplitude of p-TBK1 activation in *RB1CC1* KO +AZI2 cells (Fig. S7B). We also interrogated publicly available breast cancer patient data using KM plot [60] and found a correlation between higher levels of AZI2 expression and better relapse free survival (Figure 7E). Hence, these associations suggest that activation of AZI2-TBK1 signaling may contribute to increased CD8^+^ T cell infiltration in breast tumors and better outcomes in human breast cancer patients. Accordingly, the mechanistic insights gained through elucidation of the AZI2-TBK1 pathway could potentially have translational implications for making tumors more immunogenic in breast cancer.

## Discussion

The TBK1-IFN pathway can be activated by PAMPs (pathogen associated molecular patterns) and plays an important role as a cellular alarm system during virus or bacterial infection [61]. For that reason, it has been exploited as a strategy to make cold tumors hot and responsive to ICIs. In this study, we found a novel stimulus, whereby blockade of selective autophagy can trigger the activation of this pathway. The unique crosstalk between blockade of selective autophagy and TBK1 activation suggests that the TBK1-IFN pathway does not just respond to pathogen associated molecules but rather to pathogenic actions as well. Virophagy and bacteriophagy are primitive forms of cell autonomous immunity. However, pathogens have evolved mechanisms to inhibit selective autophagy [26,28,62] and evade degradation. It is possible that cells evolved mechanisms to counteract the inhibition of selective autophagy by pathogens, through coupling such an event to the activation of TBK1-IFN signaling. Indeed, when we overexpressed a viral protein from SARS-CoV-2 that has been reported to inhibit autophagosome-lysosome fusion (ORF3A protein) [63,64], we could observe increased phosphorylation of TBK1 (Fig. S7C). Immunofluorescence experiments also revealed increased AZI2 and p-TBK1 puncta formation in MDA-MB-231 cells expressing SARS-CoV-2 ORF3A relative to empty vector controls (Fig. S7D), suggesting that inhibition of autophagy by viral proteins can activate the AZI2-TBK1 pathway.

Another key finding from genetic dissection of the autophagy process is that the AZI2-TBK1 pathway was activated specifically upon disruption of selective autophagy but not bulk autophagy (Summarized in Figure 7F). An underlying reason for this could be the lack of involvement of AZI2 in the process of bulk autophagy. This notion is supported by our observation that HBSS induced starvation did not induce AZI2 puncta, unlike other stimuli for selective autophagy, such as FCCP and MG132 (Figures. 3A,B, Figs. S3A). Furthermore, other studies have implicated AZI2 in selective autophagy but to our knowledge, it has not been described as an essential gene for bulk autophagy [26,65]. Leveraging on the genetic mutants of RB1CC1 that are specifically deficient in bulk autophagy (*RB1CC1* KI) or selective autophagy (*RB1CC1*-deltaCT), we were able to home in on the contribution of halted selective autophagy toward AZI-TBK1 activation (Figures 3C,D and 4D–F). It would be interesting to develop these mutant alleles of RB1CC1 in mouse models to ascertain their pathological and physiological roles in future studies. Additionally, we found that genetic depletion of genes required for both types of autophagy (e.g., RB1CC1, ATG13) could induce TBK1 activation, whereas depletion of genes that were only required for LC3 lipidation and bulk autophagy (e.g., ATG5, ATG7) could not (Figures. 4A, S4B-D). It is conceivable that bulk autophagy inhibition is uncoupled from the TBK1-IFN signaling circuit, since MTOR would regularly inhibit bulk autophagy under nutrient replete conditions [32,33,66]. It is worth noting that prior studies have shown that ATG5 or ATG7 depletion can lead to elevated IFN responses [67], but one plausible explanation could be that cell specific contexts would determine the extent of IFN activation. In particular, we found that depletion of ATG5 or ATG7 could impact RB1CC1 levels as well (Figure 4A) and if RB1CC1 levels are drastically affected in a certain cell type by ATG5 or ATG7 loss, this would confound the specific contributions of the respective autophagy genes toward IFN pathway activation.

Through our analysis of the sequence of events culminating in TBK1 activation upon RB1CC1 ablation, we found that selective autophagy cargo receptors played an essential role upstream of AZI2-TBK1 (Figures. 2F–H). This is in line with the initial events that occur in the process of selective autophagy [24]. Based on our current understanding, TBK1 activation then leads to phosphorylation of cargo receptors to promote their interaction with RB1CC1 [27,38]. Hence, cells that lack RB1CC1 can still proceed through the initial steps of selective autophagy, but without phagophore formation, the cargo receptor complexes containing activated TBK1 will not be resolved, leading to accumulation in cells. Even then, TBK1 activation does not necessarily lead to downstream IFN responses, and it is well described that TBK1 adapter proteins are key mediators to achieve this [51]. In this respect, we found that AZI2 interacts with and mediates downstream activation of DDX3X, that is important for increased expression of pro-inflammatory chemokines (Figure 5). This is consistent with several studies showing that DDX3X is a substrate of TBK1 and plays a role in promoting IRF3 mediated transcription [53–56].

Apart from the mechanistic insights centered upon AZI2, we have also discovered pharmacological agents (i.e., Lys05) that can induce AZI2 puncta formation in breast cancer cells (Figure 6). Lys05 is a dimeric chloroquine molecule and is a more potent autophagy inhibitor [57], providing further evidence that pharmacological inhibition of autophagy recapitulates our findings with genetic depletion of autophagy genes (Figures 1–4). However, in addition to inhibition of autophagy in tumor cells alone, pharmacological inhibition of autophagy could also affect other components of the tumor microenvironment. It would be interesting to dissect the specific effects of autophagy inhibition in different cellular contexts for future studies. Importantly, Lys05 could also increase TBK1 activation, pro-inflammatory chemokine expression and CD8^+^ T cell infiltration (Figure 6). Nonetheless, increased CD8^+^ T cell infiltration may not necessarily lead to anti-tumor outcomes because CD8^+^ T cells can eventually become exhausted (Fig. S6E). Essentially, inhibition of autophagy through Lys05 and chloroquine derivatives could represent a potent strategy to make cold tumors hot and this could be key to making tumors responsive to ICIs [68]. In addition to activation of the TBK1 pathway, inhibition of autophagy may also confer greater benefit to ICI therapy through various other mechanisms, as observed by others [7,69–71]. Hence, it would be interesting to test chloroquine derivatives that have been refined to be more potent *in vivo* [72], as a future direction with ICIs, beyond our Lys05 observations. This would provide a foundation for translational aspects, since we observe an interesting association between higher AZI2 expression and increased CD8^+^ T cell infiltration in human breast cancer (Figures. 7A, C).

Overall, we have identified a distinct TBK1-IFN pathway that is mediated by AZI2 and responds to inhibition of selective autophagy. This pathway can promote the expression of pro-inflammatory chemokines by upregulating DDX3X activity. The insights gained suggest that autophagy inhibitors such as Lys05 could be used to activate this pathway to make cold tumors hot and responsive to ICIs.

## Materials and methods

### Reagents and antibodies

GFP-AZI2 lentiviral constructs were generated by cloning mouse AZI2 into pLenti-EFs-eGFP-Blasticidin (Gibco, A1113903) pLenti-Tet-RB1CC1-delta CT variants were generated using Q5 site directed mutagenesis kit (NEB, E0552S) according to the manufacturer’s instructions. pLV-ORF3a plasmids were a kind gift from Dr. Kefeng Lu [63] (Sichuan University, Chengdu, China). For gene silencing experiments, siRNAs used were negative control siRNA (Ambion, AM4635), human *RB1CC1* siRNA (Ambion, ID:138427), human *STING1* siRNA (Dharmacon, *M*-024333-00-0005), human *MAVS* siRNA (Dharmacon, *M*-024237-02-0005), human *DDX3X* siRNA (Dharmacon, *M*-006874-01-0005), non-target shRNA control (Sigma, SHC002) and mouse *Ddx3x* shRNA (Sigma, TRCN0000287238 and TRCN0000287239). Immunoblotting antibodies used include ACTB (Sigma, A5441), VCL (Sigma, V4505), GAPDH (Cell Signaling Technology [CST], 2118), PARP1 (CST, 9532), GFP (CST, 2555), HA (CST, 3724), ubiquitin (Santa Cruz Biotechnology, SC-8017), RB1CC1 (CST, 12436), SQSTM1 (CST, 5114), CALCOCO2 (Genetex, GTX630396), TAX1BP1 (CST, 5105), NBR1 (Genetex, GTX114539), OPTN (Proteintech, 10837–1-AP), MAP1LC3B (CST, 2775), ATG5 (CST, 12994), ATG7 (CST, 8558), phospho-TBK1 (CST, 5483), TBK1 (CST, 3504), AZI2 (Proteintech, 15042–1-AP), IRF3 (CST, 4302), p-IRF3 (CST, 4947), DDX3X (Bethyl Labs, A300-474A), p-DDX3X (Affinity Biosciences, AF3782), STAT1 (CST, 9172), p-STAT1 (CST, 9167), STING1 (CST, 13647), MAVS (CST, 3993), SQSTM1 (Enzo life Sciences, BML-PW9860) CD8A (eBioscience, 14-0808-80). Lys05 was kindly provided by Dr. Ravi Amaravadi (University of Pennsylvania, USA) [57].

### Cell culture and treatment

Primary tumor cells and their derivatives were cultured in DMEM/F12 (Gibco, 11995–065) supplemented with 10% FBS, 10 ng/mL EGF (Gibco, PMG8041), 20 mg/mL insulin (GeminiBio, 700-112P), and 50 units/mL penicillin-streptomycin. The generation of RB1CC1^f/f^;PyMT;CreER cells (Ctrl) and RB1CC1^f/KI^;PyMT;CreER cells (KI Ctrl) have been described previously [31,50] and deletion of *RB1CC1* was induced by culturing with 100 nmol/L 4-hydroxytamoxifen (4-OHT; Selleckchem, S7827) for 1 week. Transfection experiments were carried out using Lipofectamine 3000 Reagent (Invitrogen, L3000015) for cell lines. Production of lentivirus and transduction of cells were carried out as described previously [58]. For gene knockouts, sgRNA sequences used were as follows: mouse sg*Rb1cc1*: 5’-caccgCTCCATTGACCACCAGAACC-3’, mouse sg*Tbk1*: 5’-caccgCATAAGCTTCCTTCGCCCAG-3’, mouse sg*Azi2*: 5’-caccgATCTTCTACTAGCGTGTCCA-3’, mouse sg*Atg5*: 5’-caccg AAGAGTCAGCTATTTGACGT-3’ and mouse sg*Atg7*: 5’-caccgTGGACACCAGGGAGAGCCGG-3’. MDA-MB-231 and HeLa cells were obtained from ATCC (HTB-26 & CCL-2). *RB1CC1* and *ATG13* KO MDA-MB-231 cells were generated via CRISPR-CAS9 as described in our previous study [73]. HeLa Penta KO cells were a kind gift from Dr. Richard Youle (NIH) [42]. Cell lines were maintained for less than 20 passages after collection or thawing. Mycoplasma testing was performed on a monthly basis.

### Immunoblotting

Lysates were prepared from cells using modified RIPA buffer as described previously [58] with the addition of protease and phosphatase inhibitors according to manufacturer’s instructions (Thermo Scientific, PI78425 & PI78428). Protein concentrations were then quantified by bicinchoninic acid method, subjected to SDS – PAGE and analyzed by immunoblotting as described previously [58].

### Immunofluorescence, histology and immunohistochemistry

Immunofluorescence analysis of cells were performed as described previously [74] and analyzed using a Zeiss LSM710 confocal microscope with a Zeiss AxioObserver Z1 stand. Formalin-fixed paraffin-embedded (FFPE) tumors were sectioned with a thickness of 5 µm and stained for respective antigens as described previously [58]. Antigen retrieval was performed in citrate buffer with a pressure cooker. Breast cancer tissue microarray slides (TMA BC01) were purchased from Reveal Biosciences.

### Flow cytometry and imaging cytometry

Single-cell suspensions were prepared from tumor cells as described previously [75]. Cells were then sorted using a FACSAria instrument (BD Biosciences). Flow cytometry data were analyzed using FlowJo software. For imaging cytometry, cells were analyzed using Amnis Image Stream MK II imaging flow cytometer (Luminex).

### Liquid chromatography- Mass spectrometry (LC-MS) identification of AZI2 interacting proteins

Mass spectrometry experiments for identification of AZI2 interacting proteins were performed by the UC Proteomics Core. GFP-AZI2 or GFP were precipitated from respective Ctrl or *RB1CC1* KO cells using GFP-TRAP magnetic agarose beads (Chromotek, GTMA010) according to manufacturer’s instructions. Immunoprecipitated proteins in 2X Laemmli buffer were run into Invitrogen 4–12% BT gels using MOPS buffer for 1.5 cm. The sample lanes were excised, reduced with DTT, alkylated with IAA, and digested overnight with Trypsin. The peptides were extracted, dried, and resuspended in 0.1% Formic acid (FA). After centrifugation at 10,000 × g to remove particulates, the samples were transferred to an autosampler vial where each IP sample was analyzed by nano LC-MS/MS (Orbitrap Eclipse). The LC-MS/MS results were searched against mouse UniProt database (UP000000589) using Proteome Discoverer ver 2.4 and the Sequest HT search algorithm (Thermo scientific).

### Inhibitor screen for AZI2 puncta inducing compounds

GFP-AZI2 -4OHT cells were seeded in 24-well plates and left overnight before treatment with respective inhibitors. Upon treatment, images of cells were acquired every 2 h with an Incucyte live-cell imaging system (Essen Bioscience) to monitor GFP-AZI2 puncta formation. Inhibitor concentrations used in this screen were; SB203580, 10 µM (MedChemExpress, HY-10256); Tozasertib, 5 µM (MedChemExpress, HY-10161); Chloroquine, 100 µM (Sigma Aldrich, C6628); GDC0994, 5 µM (Cayman Chemical, 21107); Prexasertib, 5 µM (MedChemExpress, HY-18174); LTURM34, 10 µM (MedChemExpress, HY-101667); GSK650394, 5 uM (MedChemExpress, HY-15192); Amlexanox, 100 µM (MedChemExpress, HY-B0713); U0126, 10 µM (Cayman Chemical, 70970); IRAK 1–4 Inhibitor I, 10 µM (MedChemExpress, HY-13329); SP600125, 25 µM (MedChemExpress, HY-12041); SBI0206965, 10 µM (Cayman Chemical, 18477); BMS345541, 10 µM (MedChemExpress, HY-10519); Triciribine, 10 µM (MedChemExpress, HY-15457); Silmitasertib, 10 µM (MedChemExpress, HY-50855); PP242, 5 µM (MedChemExpress, HY-10474); Lys05, 20 µM (provided by Dr. Ravi Amaravadi); Palbociclib, 5 µM (MedChemExpress, HY-50767); and D4476, 50 µM (MedChemExpress, HY-10324). The puncta/image at 24 h were quantified and plotted.

### Tumor mice and transplants

Mice were housed and handled according to local, state, and federal regulations. All experimental procedures were carried out according to protocols approved by the Institutional Animal Care and Use Committee at University of Cincinnati (Cincinnati, OH). For transplantation experiments, 2 × 10^6^ iKO cells were prepared in PBS:Matrigel at a 1:1 ratio and were injected into the fourth mammary gland fat pad. Mice with transplanted tumors were randomized into respective treatment groups when the diameter of tumors reached ~5 mm. For Lys05 treatments, mice were treated with 50 mg/kg of Lys05 or PBS as vehicle control, intraperitoneally (i.p.), once daily for indicated times.

### Quantitative PCR

GeneGet RNA Purification Kit (Thermo Scientific, K0731) was used to isolate total RNA from cells according to manufacturer’s instructions. RNA (equal amounts) were then reverse transcribed using iScript cDNA Synthesis Kit (Bio-rad, 1708891). qRT-PCR analysis was performed using iTaq Universal SYBR Green Supermix (Bio-rad, 1725121), with the respective primers: *Ifna*-Forward; 5’- CCACAGGATCACTGTGTACCTGAGA-3’, *Ifna*-Reverse; 5’- CTGACCACCTCCCAGGCACAG-3’, *Ifnb*-Forward; 5’- AAGAGTTACACTGCCTTTGCCATC-3’, *Ifnb*-Reverse; 5’- CACTGTCTGCTGGTGGAGTTCATC-3’, *Actb*-Forward; 5’-GGCTGTATTCCCCTCCATCG-3’, *Actb*-Reverse; 5’-CCAGTTGGTAACAATGCCATGT-3’, *Ccl5*-Forward; 5’-GCTGCTTTGCCTACCTCTCC-3’, *Ccl5*-Reverse; 5’-TCGAGTGACAAACACGACTGC-3’, *Cxcl10*-Forward; 5’-CCAAGTGCTGCCGTCATTTTC-3’, *Cxcl10*-Reverse; 5’-GGCTCGCAGGGATGATTTCAA-3’, *Cxcl9*-Forward; 5’-GGAGTTCGAGGAACCCTAGTG-3’, *Cxcl9*-Reverse; 5’-GGGATTTGTAGTGGATCGTGC.

### Statistical analysis

Datapoints were plotted as means with standard error (SEM). One-way comparisons were tested using unpaired t-test and for multiple comparisons, one-way ANOVA test was performed. For correlation analysis of AZI2 and CD8 scores in human breast cancers, Pearson and Spearman tests were performed. The threshold for statistical significance was *p* < 0.05.

## Supplementary Material

Supplemental Material

Supplemental Material

## Data Availability

All relevant data to evaluate the conclusions in the paper are within the paper and/or the Supplementary Materials.
