# Supplementary material for: AZI2 mediates TBK1 activation at unresolved selective autophagy cargo receptor complexes with implications for CD8 T-cell infiltration in breast cancer
Source: Autophagy. 2023 Sep 21;20(3):525–40. doi: 10.1080/15548627.2023.2259775 (PMC10936636; doi:10.1080/15548627.2023.2259775)
Supplement: Supplemental Material [file KAUP_A_2259775_SM4714.docx]

**
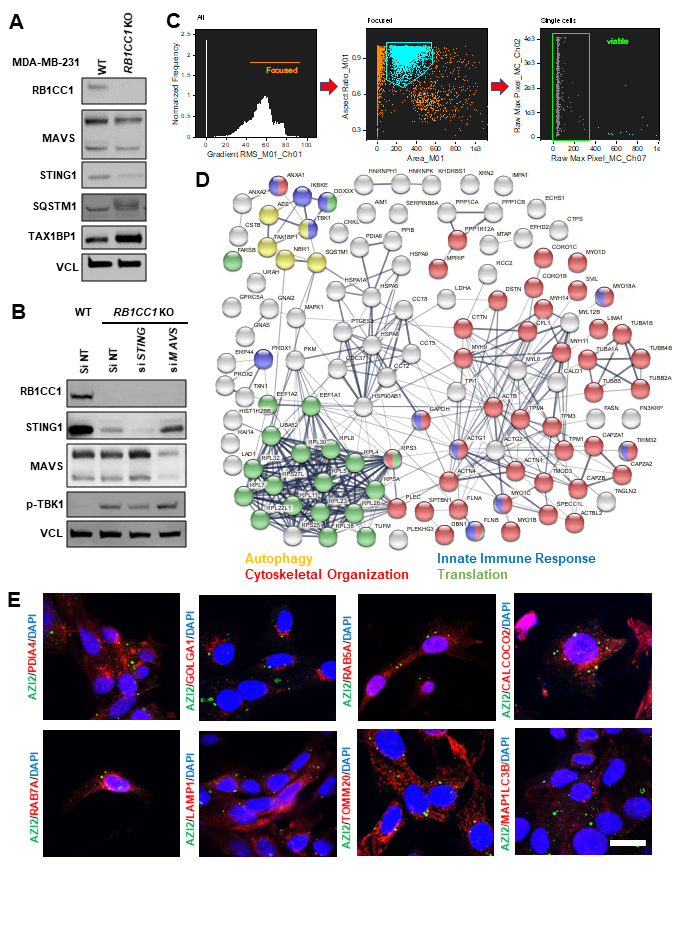
**

**Figure S1.** AZI2 puncta formation in *RB1CC1* KO cells. (**A**) Immunoblots showing levels of RB1CC1, MAVS, STING, SQSTM1, TAX1BP1 and VCL in WT and *RB1CC1* KO MDA-MB-231 cells. (**B**) Immunoblots showing levels of RB1CC1, MAVS, STING1, p-TBK1 and VCL in WT and *RB1CC1* KO MDA-MB-231 cells treated with non-targeting siRNA (siNT), *STING1* siRNA (si *STING1*) or *MAVS* siRNA (si *MAVS*). (**C**) Gating strategy for imaging cytometry analysis of cells with GFP-AZI2 puncta. (**D**) STRING network analysis of GFP-AZI2-interacting proteins in *RB1CC1* KO +AZI2 cells from mass spectrometry data. (**E**) Confocal imaging of GFP-AZI2 and other markers as indicated, in *RB1CC1* KO +AZI2 cells. Scale bar: 10 µm.

**
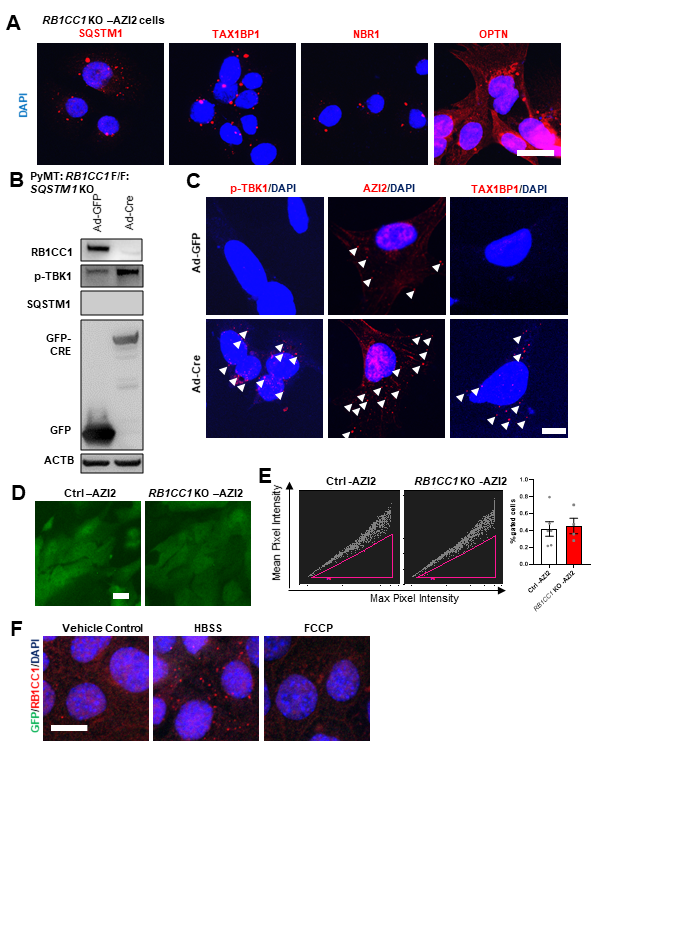
**

**Figure S2.** Cargo receptor puncta formation in *RB1CC1* KO cells. (**A**) Confocal imaging of SQSTM1, TAX1BP1, NBR1 or OPTN in *RB1CC1* KO -AZI2 cells. Scale bar: 10 µm. (**B**) Immunoblots showing levels of RB1CC1, p-TBK1, SQSTM1, GFP or ACTB in PyMT: *RB1CC1* F/F: *SQSTM1* KO cells transduced with adenovirus encoding Ad-GFP or Ad-Cre. (**C**) Confocal imaging of p-TBK1, AZI2 or TAX1BP1 in cells described in B. Scale bar: 5 µm. **(D)** Confocal imaging of GFP in Ctrl -AZI2 and *RB1CC1* KO -AZI2 cells. Scale bar: 10 µm. (**E**) Dot plots showing mean pixel intensity against max pixel intensity from imaging cytometry analysis of Ctrl or *RB1CC1* KO -AZI2 cells. Bar chart shows percentage gated cells representing cells with minimal GFP puncta. (**F**) Confocal imaging showing AZI2 (green) and RB1CC1 (red) puncta formation in Ctrl -AZI2 cells cultured under control, HBSS conditions, or 20 µM FCCP treatment for 3 h. Scale bar: 10 µm.

**
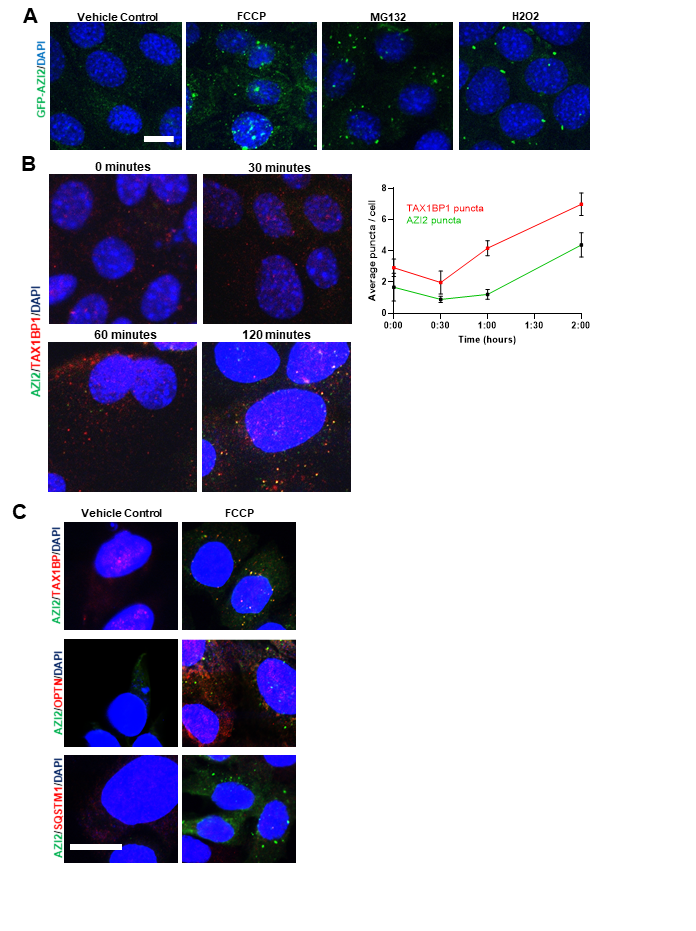
**

**Figure S3.** AZI2 puncta and cargo receptor puncta formation. (**A**) Confocal imaging of GFP-AZI2 in Ctrl +AZI2 cells treated with vehicle, 20 µM FCCP, 10 µM MG132 or 1 mM H_2_O_2_ for 3 h. Scale bar: 10 µm. (**B**) Confocal imaging of GFP-AZI2 (green) and TAX1BP1 (Red) in Ctrl +AZI2 cells treated with 40 µM FCCP for the indicated amount of time. Line graph showing quantification of TAX1BP1 or AZI2 puncta formation over time. **(C)** Confocal imaging showing AZI2 (green) and cargo receptor (red) puncta formation in Ctrl +AZI2 cells treated with 20 µM FCCP treatment for 3 h. Scale bar: 10 µm.

**
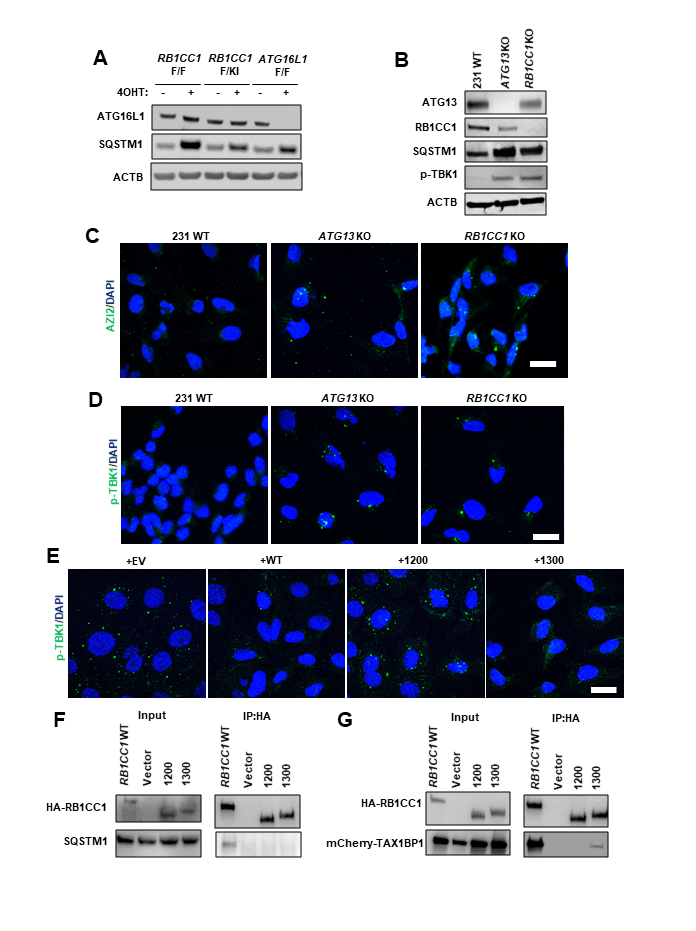
**

**Figure S4.** Inhibition of selective autophagy but not bulk autophagy leads to TBK1 activation. **(A**) Immunoblots showing levels of ATG16L1, SQSTM1 or ACTB in *RB1CC1* F/F, *RB1CC1* F/KI or *ATG16L* F/F cells treated with or without 4-OHT to induce deletion of floxed genes. (**B**) Immunoblots showing levels of ATG13, RB1CC1, SQSTM1, p-TBK1 or ACTB in MDA-MB-231 WT, *ATG13* KO or *RB1CC1* KO cells. (**C-D**) Confocal imaging of (**C**) AZI2 and (**D**) p-TBK1 in MDA-MB-231 WT, *ATG13* KO or *RB1CC1* KO cells. Scale bar: 20 µm. (**E**) Confocal imaging of p-TBK1 in *RB1CC1* KO cells transduced with doxycycline inducible empty vector, RB1CC1 WT, RB1CC1 residues 1-1200 or RB1CC1 residues 1-1300. Scale bar: 10 µm. (**F**) Immunoblots showing input and immunoprecipitation (IP:HA) lysates from HEK293 cells transfected with *RB1CC1* constructs and *SQSTM1*. **(G)** Immunoblots showing input and immunoprecipitation (IP:HA) lysates from HEK293 cells transfected with *RB1CC1* constructs and *TAX1BP1*.


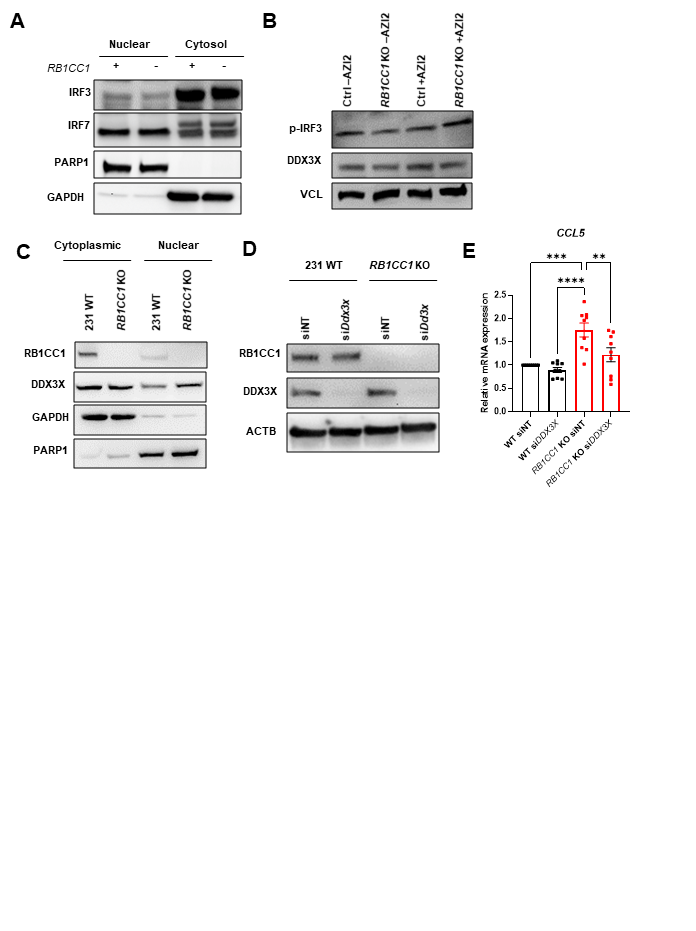


**Figure S5.** DDX3X as a downstream effector of AZI2-TBK1 activation. (**A**) Immunoblots showing levels of IRF3, IRF7, PARP1 and GAPDH in nuclear and cytoplasmic enriched protein lysates from Ctrl and *RB1CC1* KO cells. (**B**) Immunoblots showing levels of p-IRF3, DDX3X and VCL in Ctrl -AZI2, *RB1CC1* KO -AZI2, Ctrl +AZI2 and *RB1CC1* KO +AZI2 cells. (**C**) Immunoblots showing levels of RB1CC1, DDX3X, GAPDH and PARP1 in nuclear and cytoplasmic enriched protein lysates from MDA-MB-231 WT or *RB1CC1* KO cells. (**D**) Immunoblots showing levels of RB1CC1, DDX3X and ACTB in MDA-MB-231 WT or *RB1CC1* KO cells treated with siNT or si*DDX3X*. (**E**) Bar chart showing levels of the chemokine *CCL5* in MDA-MB-231 cells as described in D.

**
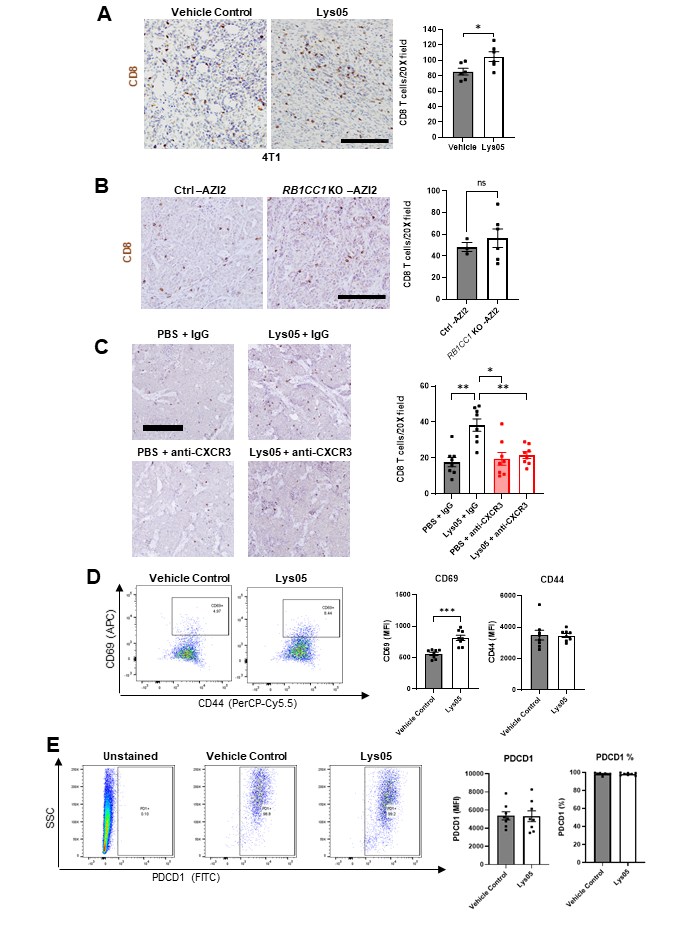
**

**Figure S6.** AZI2 dependent recruitment of CD8^+^ T cell into tumors upon autophagy blockade. (**A**) Micrographs showing levels of CD8^+^ T cell infiltration for PBS or Lys05 treated mice bearing 4T1 syngeneic tumors. Scale bar: 200 µm. Bar chart shows quantification of CD8^+^ T cells per 20x field of view, * indicates p<0.05. (**B**) Micrographs showing levels of CD8^+^ T cell infiltration in mice bearing Ctrl -AZI2 or *RB1CC1* KO -AZI2 syngeneic tumors. Scale bar: 200 µm. Bar chart shows quantification of CD8^+^ T cells per 20x field of view, ns indicates not statistically significant. (**C**) Micrographs showing levels of CD8^+^ T cell infiltration for PBS + IgG, Lys05 + IgG, PBS + anti-CXCR3 or Lys05 + anti-CXCR3 treated mice bearing PyMT syngeneic tumors. Scale bar: 200 µm. Bar chart shows quantification of CD8^+^ T cells per 20x field of view, * indicates p<0.05, ** indicates p<0.01. **(D)** Dotplots showing levels of CD69 and CD44 in CD8^+^ T cell populations. Bar charts show quantification of percentage of cells expressing CD69 or CD44 among CD8^+^ T cell populations. **(E)** Dotplots showing levels of PDCD1/CD279/PD-1 in CD8^+^ T cell populations. Bar charts show quantification of mean fluorescence intensity (MFI) and percentage of cells for PDCD1 in CD8^+^ T cell populations.

**
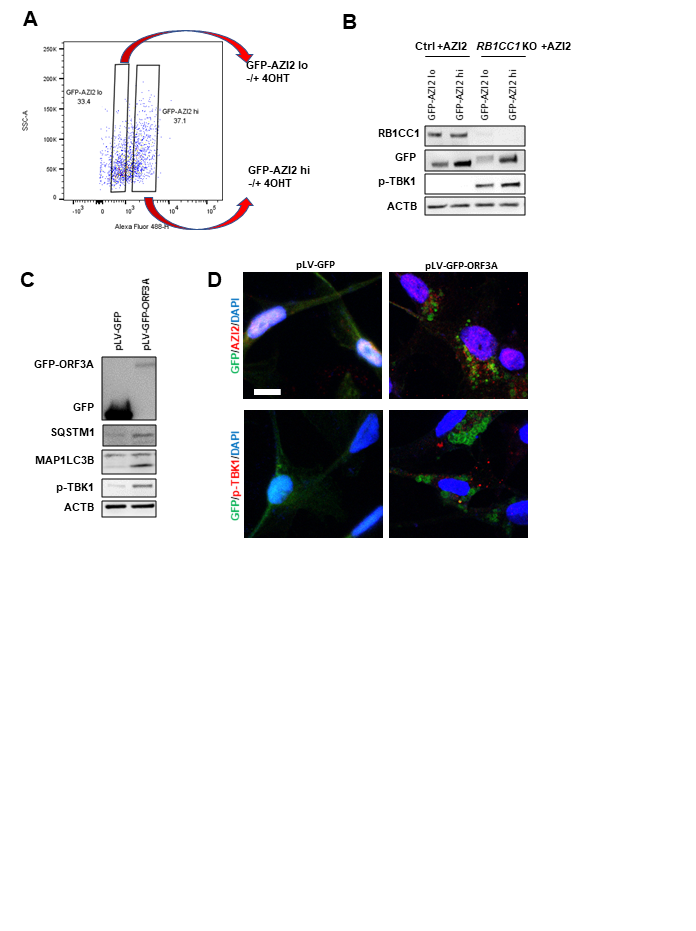
**

**Figure S7.** AZI2 levels determine magnitude of TBK1 activation and AZI2-TBK1 pathway activation by SARS-CoV2 ORF3A protein. (**A**) Dot plot showing sorting gates for GFP-AZI2 hi and GFP-AZI2 lo cell populations. (**B**) Immunoblots showing levels of RB1CC1, GFP, p-TBK1 and ACTB in GFP-AZI2 hi and GFP-AZI2 lo populations from Ctrl +AZI2 and *RB1CC1* KO +AZI2 cells. (**C**) Immunoblots showing levels of GFP, SQSTM1, MAP1LC3B, p-TBK1 and ACTB in MDA-MB-231 cells transduced with control vector or GFP-ORF3A expression vector. (**D**) Confocal imaging of GFP along with AZI2 or p-TBK1 in cells described in C. Scale bar: 10 µm.
